# Effects of Fibrin Glue on Postoperative Complications After Modified Radical Mastectomy for Breast Carcinoma

**DOI:** 10.7759/cureus.115225

**Published:** 2026-08-26

**Authors:** Sriya Srujana Srujana, Ludia John, Aditi Gopinath, Narayanan Cunnigaiper

**Affiliations:** 1 General Surgery, Sri Ramachandra Institute of Higher Education and Research, Chennai, IND

**Keywords:** breast cancer, drain removal, earlier recovery and discharge, fibrin glue, flap necrosis, mrm, seroma, wound dehiscence

## Abstract

Background

Seroma formation is a common postoperative complication following modified radical mastectomy (MRM) for breast cancer, contributing to delayed wound healing, increased morbidity, prolonged hospitalization, and delay in adjuvant treatment. Fibrin glue, a biological adhesive, is proposed to reduce seroma formation by sealing lymphatic channels and reducing dead space. This study evaluated the effectiveness of fibrin glue combined with closed suction drainage in reducing postoperative complications following MRM compared to suction drainage alone.

Methodology

A prospective observational study was conducted at a tertiary care center from March 2024 to September 2025. Patients undergoing MRM were divided into the following two groups: Group A (fibrin glue + suction drain) and Group B (suction drain only). Postoperative parameters including drain output, pain scores, seroma formation, wound complications, and hospital stay were recorded. Statistical analysis was performed using the Mann-Whitney U test and chi-square test, with p-values <0.05 considered significant.

Results

The mean age was 55.58 ± 10.01 years in Group A and 54.50 ± 8.08 years in Group B (p = 0.800). Mean body mass index was 26.89 ± 5.14 kg/m² and 27.03 ± 4.48 kg/m², respectively (p = 0.832). Postoperative drain output on postoperative day (POD) one was significantly lower in the fibrin glue group (28.07 ± 17.93 mL) compared to the control group (51.15 ± 7.31 mL; p = 0.001), with similar significant differences noted through POD seven. Total drain output was 251.95 ± 236.22 mL in Group A versus 522.10 ± 260.18 mL in Group B (p = 0.003). Drain removal was earlier in the fibrin group (3.16 ± 0.37 days vs. 5.90 ± 1.59 days; p < 0.0001). Seroma occurred in 0% of Group A and 5.26 % of Group B patients (p = 0.299). Mean hospital stay was 4.36 ± 1.11 days in Group A and 10.50 ± 2.64 days in Group B (p < 0.0001). Wound dehiscence was observed only in the non-fibrin group (10.5%).

Conclusions

Fibrin glue significantly reduced total drain output, time to drain removal, and hospital stay. Although the reduction in seroma incidence was not significant, the overall clinical outcomes favor fibrin glue as an effective adjunct in post-MRM management.

## Introduction

Breast cancer is the most diagnosed cancer in women worldwide and ranks second among all cancers in both men and women, with more than 2.3 million new cases each year [1]. Modified radical mastectomy (MRM), which removes the whole breast along with axillary lymph nodes, is widely used as one of the modalities for the treatment of invasive mammary carcinoma, as appropriate [2]. Although the procedure is generally effective for local disease control, it often results in postoperative complications, the most prevalent of which is seroma formation [3].

Seroma is the build-up of clear fluid in the empty space left after tissue has been surgically removed, especially beneath mastectomy flaps or within the axilla. Reports suggest that seromas occur in approximately 15-81% of cases following mastectomy, making them the most frequent postoperative complication in this context [4]. Although often considered a self-limiting surgical outcome, persistent or voluminous seromas can contribute to surgical site infection, delayed wound healing, wound dehiscence, skin flap necrosis, and extended hospital stays, thus complicating the overall recovery process [5].

Various strategies have been employed to address seroma formation, including suction drainage, quilting sutures, external compression, sclerosing agents, and tissue sealants [6]. Among these, fibrin glue has emerged as a promising treatment modality. Composed primarily of fibrinogen and thrombin, fibrin glue mimics the final step of the coagulation cascade, resulting in the formation of a fibrin clot. This biological sealant serves multiple purposes: it enhances hemostasis, promotes tissue adhesion, and potentially obliterates the dead space that contributes to seroma accumulation [7].

However, not all studies have reported clear benefits [8,9]. These inconsistencies underscore the need for further well-designed controlled studies to clarify the contexts in which fibrin glue may be most beneficial. Factors such as the volume of glue used, method of application, body mass index (BMI), extent of dissection, and surgical technique may influence outcomes. However, the predominance of favorable findings positions fibrin glue as a valuable adjunct in minimizing seroma formation after MRM, potentially leading to faster recovery and reduced healthcare resource utilization.

This study was conducted to assess whether using fibrin glue along with closed-suction drainage is more effective than using closed-suction drainage alone in reducing seroma formation after MRM for breast cancer.

## Materials and methods

This single-center, prospective, comparative observational study was conducted in the Department of General Surgery at a tertiary care center in South India between March 2024 and September 2025. Approval was obtained from the Institutional Ethics Committee (approval number: CSP-MED/23/SEP/93/219), and written informed consent was obtained from all the participants who met the inclusion criteria.

All women over the age of 18 years who were diagnosed with invasive mammary carcinoma and undergoing MRM were included in the study. Patients who had previous surgery or radiation on the operated side, BMI of more than 35 kg/m², and those on steroids and anticoagulants were excluded from the study.

During the study period, the patients who were planned for MRM and satisfied the inclusion and exclusion criteria were included in the study. After informed consent was obtained, those patients who were willing to receive fibrin glue application were allocated to the fibrin glue arm, and others to the control arm. At the end of the study period, 20 patients received fibrin glue, and 19 patients received no fibrin glue (control group). Standard perioperative care protocols were followed in both groups.

The outcomes measured included seroma, drain output, time to discharge, wound dehiscence, and flap necrosis. Seroma was defined as postoperative fluid collection at the surgical site. Flap necrosis, defined as death of tissue within a surgical flap, was detected as blackish discoloration at the flap edges, while wound dehiscence was detected as partial or complete separation of the wound edges.

Surgical steps

All patients underwent MRM (Auchincloss method) using a standardized surgical technique, with an elliptical incision over the breast encompassing the nipple-areolar complex. Skin flaps were elevated by dissecting through the plane separating the subcutaneous tissue from the underlying mammary gland. Dissection continued superiorly toward the clavicle, medially toward the sternum, inferiorly into the inframammary region, and laterally to the anterior border of the latissimus dorsi muscle. The entire breast was then mobilized with the pectoral fascia. The clavipectoral fascia was incised, and the plane between and beneath the major and minor pectoral muscles was carefully dissected while preserving the medial pectoral nerve. The axillary vein was visualized, and its first major branch was ligated at its junction with the chest wall. The thoracodorsal and long thoracic nerves were identified and preserved throughout. Complete excision of Level I and Level II and Level III (if indicated) axillary lymph nodes followed, and the specimen was detached by transecting the lateral extension of subcutaneous adipose tissue. Electrocautery was used sparingly to minimize thermal damage, and careful hemostasis was maintained in all cases, followed by placement of two closed-suction drains, one beneath the skin flaps and one within the axilla.

For the intervention group, 4 mL of fibrin glue was prepared intraoperatively (2 mL for the chest wall and 2 mL for the axilla), diluted per manufacturer guidelines. During closure of the subcutaneous tissue, two gaps were left, one over the chest wall and another over the axilla. The glue was sprayed onto the flaps immediately before closure using an aerosol device, and gentle uniform pressure was maintained on the chest wall for five minutes. The skin was sutured closed. A soft cotton pad was placed over the axilla and chest wall, and a pressure dressing was applied.

In the control group, the same surgical technique was followed, including careful hemostasis, but fibrin glue was not used. Drains were placed as described above, and the flaps closed immediately.

In both groups, drain output was monitored daily on a 24-hour basis. Drains were removed once output fell to ≤30 mL/day for three consecutive days. Patients were assessed daily until discharge from hospital and reassessed on postoperative day (POD) 7 and day 30 for seroma formation and seroma-related flap necrosis. All the details were meticulously entered into a proforma.

Statistical analysis

Data are presented as frequencies, percentages, medians, and interquartile ranges. The Mann-Whitney U test was used to compare continuous variables, and the independent-samples t-test or Pearson’s chi-square test was used for categorical variables. A two-tailed p-value <0.05 was considered significant. Statistical analysis was performed using SPSS version 21.0 (IBM Corp., Armonk, NY, USA).

## Results

A total of 39 patients were included in this study, with 20 patients in the fibrin glue group and 19 patients in the comparator control group. The mean age was 55.58 ± 10.01 years in patients treated with fibrin glue and 54.50 ± 8.08 years in those managed without it. Baseline characteristics were similar between cohorts. The mean BMI was 26.89 ± 5.14 kg/m² for the fibrin group and 27.03 ± 4.48 kg/m² for the control group (p = 0.832). Comorbidities in the fibrin versus non-fibrin groups included diabetes (31.6% vs. 20.0%) and hypertension (42.1% vs. 15.0%), while bronchial asthma occurred in 5 % of the control group. None of these differences between the two groups reached statistical significance.

On POD one, mean drain output was 28.07 ± 17.93 mL in the fibrin glue group versus 51.15 ± 7.31 mL in the non-fibrin group. On POD two, output was 22.11 ± 17.84 mL versus 38.75 ± 14.64 mL; on POD three, 20.05 ± 16.29 mL versus 32.05 ± 8.34 mL; on POD four, 22.69 ± 3.12 mL versus 31.85 ± 10.14 mL; and on POD five, 25.00 ± 10.46 mL versus 27.47 ± 5.87 mL. On POD six and POD seven, the fibrin glue group recorded 23.62 ± 7.86 mL and 31.31 ± 8.76 mL, while the non-fibrin group recorded 23.20 ± 8.93 mL and 33.40 ± 10.14 mL, respectively. Mann-Whitney U test for differences in postoperative drain output between the groups showed significant differences from POD one to seven (p = 0.001, 0.001, 0.002, 0.004, 0.002, 0.004, and <0.0001, respectively) (Table 1).

**Table 1:** Comparison of postoperative drain output (days 1 to 11) between fibrin glue and control groups.

Postoperative day	Fibrin glue group (mean ± SD, mL)	Non-fibrin group (Mean ± SD, mL)	P-value (Mann-Whitney U test)
1	28.07 ± 17.93	51.15 ± 7.31	0.001
2	22.11 ± 17.84	38.75 ± 14.64	0.001
3	20.05 ± 16.29	32.05 ± 8.34	0.002
4	22.69 ± 3.12	31.85 ± 10.14	0.004
5	25.00 ± 10.46	27.47 ± 5.87	0.002
6	23.62 ± 7.86	23.20 ± 8.93	0.004
7	31.31 ± 8.76	33.40 ± 10.14	<0.0001
8	31.08 ± 10.19	26.25 ± 3.10	1.000
9	28.77 ± 8.49	26.75 ± 3.95	0.092
10	29.67 ± 11.78	29.00	0.305
11	29.08 ± 8.93	30.00	0.400

**Figure 1:**
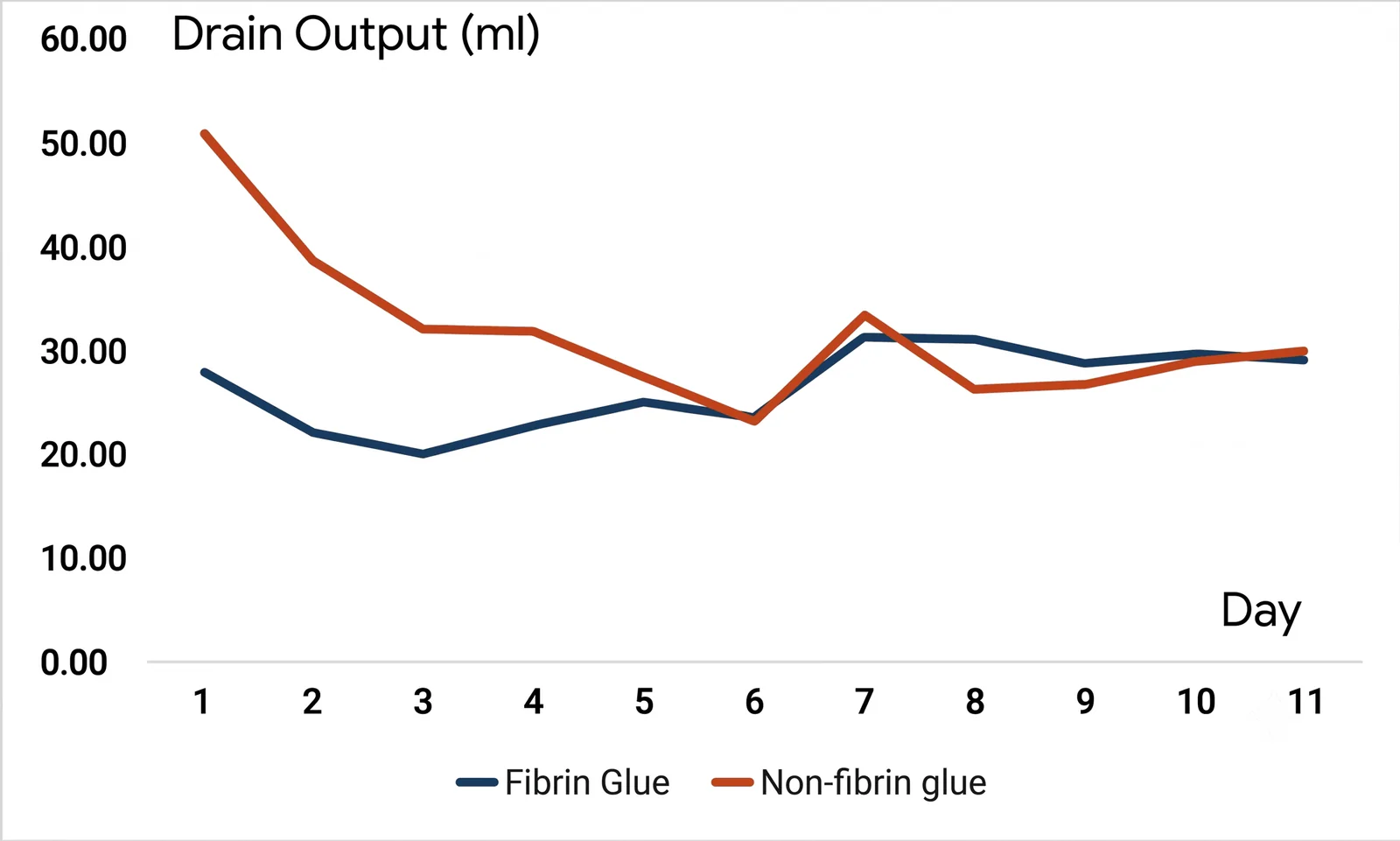
Comparison of daily postoperative drain output.

From POD eight through 11, no significant differences were observed: POD eight, 31.08 ± 10.19 mL (fibrin) vs. 26.25 ± 3.10 mL (non-fibrin); POD nine, 28.77 ± 8.49 mL vs. 26.75 ± 3.95 mL; POD 10, 29.67 ± 11.78 mL vs. 29.00 mL; POD 11, 29.08 ± 8.93 mL vs. 30.00 mL (p = 1.000, 0.092, 0.305, and 0.400, respectively) (Figure 1).

The hospital stay was 4.36 ± 1.11 days in patients who received fibrin glue and 10.50 ± 2.64 days in those who did not, with a significant difference using the independent-samples t-test (p < 0.0001).

Seroma occurred in none of the patients treated with fibrin glue, compared with 5.26 % of those managed without it; using the chi-square test, this difference was not statistically significant (p = 0.299) (Table 2).

**Table 2:** Comparison of seroma formation.

Seroma formation	Fibrin glue		Non-fibrin glue		P-value (chi-square test)
	Count	Percentage (%)	Count	Percentage (%)	
Yes	0	0.00	1	5.26	0.299 (χ² = 1.081, df = 1)
No	20	100.00	18	94.74	

Chest wall drain removal occurred at 3.16 ± 0.37 days with fibrin glue versus 5.90 ± 1.59 days without; for axillary drain removal, the corresponding durations were 4.32 ± 1.11 and 10.50 ± 2.65 days. This difference in timing was significant for both drain locations as determined by the independent-samples t-test (Table 3).

**Table 3:** Comparison of drain removal time.

Drain removal	Fibrin glue		Non-fibrin glue		P-value (t-test)
	Mean (mL)	SD (mL)	Mean (mL)	SD (mL)	
Chest wall drain	3.16	0.37	5.90	1.59	<0.0001 (t = -7.50, df = 37)
Axillary drain	4.32	1.11	10.50	2.65	<0.0001 (t = -9.59, df = 37)

By POD seven, flap necrosis occurred in 5.0% of patients treated with fibrin glue, compared with 10.52% of those in the non-fibrin group (p = 0.605). Seroma was absent in the fibrin cohort but noted in 5.26% of the non-fibrin cohort (p = 0.487). Wound healing was satisfactory in 95% of fibrin glue-treated cases and 84.21% of non-fibrin cases. On Fisher’s exact test, overall wound status on day seven showed no significant difference between groups (p = 0.367).

On POD 14, none of the patients who managed with fibrin glue developed seroma or flap necrosis. Total postoperative drain output was 251.95 ± 236.22 mL with fibrin glue versus 522.10 ± 260.18 mL with a statistically significant reduction using the independent-samples t-test (p = 0.003) (Table 4).

**Table 4:** Comparison of postoperative complications in the fibrin glue and non-fibrin glue group.

Complication	Fibrin glue (N = 20)		Non-fibrin glue (N = 19)		P-value (Fisher’s exact test)
	Count	Percentage (%)	Count	Percentage (%)	
Seroma	0	0.00	1	5.26	0.487
Flap necrosis	1	5.00	2	10.52	0.605
Wound dehiscence	0	0.00	2	10.52	0.231

## Discussion

Increasing evidence supports the role of fibrin glue in lowering seroma formation after MRM. In a prospective observational study, Sharma et al. showed that applying fibrin glue during surgery significantly reduced drain output, seroma size, and the length of time drains remained, compared to using suction drains alone with standard closure [10]. Similarly, Tolba et al., in a randomized study involving 30 patients, reported that those who received fibrin glue exhibited reduced fluid accumulation, shorter hospital stays, and earlier drain removal [11]. A randomized controlled trial by Faisal et al. highlighted a significant reduction in total drain output and time to drain removal in patients receiving autologous fibrin glue versus controls, with cumulative drain volumes of 505.6 ± 209.3 mL and 1,674.1 ± 1,373.8 mL, respectively [12]. A similar pattern was observed in a trial by Azhar et al., who found a significant reduction in seroma volume and hospital stay among patients treated with autologous fibrin glue [13]. However, certain studies did not arrive at similar conclusions. In a Swedish trial, Udén et al. found no significant difference in seroma occurrence between fibrin glue and control groups, suggesting that factors such as surgical skill and technique may affect results [8]. Likewise, Ulusoy et al. observed no meaningful benefit in terms of seroma formation or duration of drainage with the use of fibrin glue [9].

In our study, the fibrin glue and non-fibrin glue cohorts had comparable demographic and baseline characteristics, with no notable differences in age, BMI, clinical stage, or comorbid conditions, ensuring a balanced comparison before surgery with elimination of confounding factors. The use of fibrin glue significantly reduced postoperative drain output, particularly during the first week following surgery. The fibrin glue cohort exhibited a substantial decrease in total postoperative drainage, recording 251.95 mL compared to 522.10 mL in the control group (p = 0.003). This effect was most pronounced from POD one to seven; for example, on POD one, the fibrin group averaged 28.07 mL while the non-fibrin group averaged 51.15 mL (p = 0.001). These results are consistent with prior studies demonstrating significantly reduced drainage volumes with the use of fibrin glue [11,13,14]. For example, Azhar et al. [13] reported a total output of 516 mL versus 1,164 mL in controls (p = 0.0001), and Tolba et al. noted a decrease from 1,340 mL to 593.33 mL (p < 0.001) [11]. While highly effective for early fluid control, the data indicate that differences in drainage volumes between the two groups generally become statistically insignificant after the seventh day [11,13,14].

The use of fibrin glue significantly improved patient recovery by enabling earlier drain removal, with averages ranging from 3.16 to 4.32 days compared to 5.90 to 10.50 days in control groups (p < 0.0001). This clinical efficiency is directly supported by the glue’s ability to reduce total postoperative drainage to 251.95 mL from 522.10 mL (p = 0.003). This resulted in decreased hospital stay, averaging 4.36 days for the fibrin group versus 10.50 days for the non-fibrin group (p < 0.0001), a finding corroborated by external studies showing stays as short as 1.35 to 3.6 days. Earlier drain removal in the fibrin group did not compromise wound healing or increase complications. While most research aligns with these benefits [12,15], some variability exists, such as Mustonen et al. reporting no significant difference in recovery duration [16].

Tolba et al. (2023) and Faisal et al. (2021) both reported significantly lower seroma rates, and Fawzy et al. (2017) observed a reduction from 35.0% to 5.0% [11,12,17]. In our study, seroma was absent in the fibrin glue group but occurred in 5.26% of patients without fibrin glue (p = 0.299). Flap necrosis was seen in 5.0% of the fibrin group compared to 10.5% in the non-fibrin group (p = 0.517). No cases of wound dehiscence were reported in fibrin-treated patients, whereas 10.5% of those without fibrin glue experienced this complication (p = 0.136). On POD seven, seroma appeared in 5.26% of patients in the non-fibrin group but was not observed in the fibrin group. By POD 14, one patient in the non-fibrin group required revision surgery due to seroma and flap necrosis. While these differences were not significant, the results indicate that fibrin glue may contribute to reducing early postoperative complications.

Limitations

The limitations of the study include a limited sample size and its single-center design, which may restrict the generalizability of the findings. The absence of randomization and blinding introduces potential selection bias and residual confounding. Only patients undergoing elective MRM were included, excluding those with neoadjuvant therapy or immediate reconstruction. The small number of events limits reliable comparison of postoperative complications, and the statistical analysis of repeated drain output measurements requires further consideration. The short follow-up period focused on early outcomes, without assessing long-term complications. Minor variations in surgical technique and fibrin glue application may have influenced results. In our study, it was noted that patients with lower BMI and smaller breasts had decreased drain output within the fibrin group. Hence, we hypothesize that more volume of fibrin glue is required in patients with increased BMI and larger breasts for better efficacy of fibrin glue application. However, this association was not quantified. Cost is a significant factor for procurement and regular use of fibrin glue in lower- and middle-income countries.

Future recommendations

Multicenter trials with larger sample sizes and long-term follow-ups are recommended. More studies that adjust the volume of fibrin glue according to BMI and breast volume are required to determine whether the volume of fibrin glue used is a limiting factor in its effectiveness in large-volume breasts.

## Conclusions

This study showed that the application of fibrin glue during MRM for breast cancer significantly lowers postoperative drain output, shortens the time to drain removal, and allows earlier discharge. Although the decrease in seroma rates was not statistically significant, the absence of seroma in the fibrin group and fewer complications suggest a probable benefit that needs to be corroborated with larger randomized controlled trials. These results suggest that fibrin glue is a useful adjunct for improving outcomes after MRM; wider use could reduce patient morbidity and ease the burden on healthcare resources. However, larger randomized controlled trials are necessary to confirm its routine use and cost-effectiveness in breast cancer surgery. This study should be taken into consideration for future studies involving larger sample sizes and comparator groups.
